# The use of expert elicitation in environmental health impact assessment: a seven step procedure

**DOI:** 10.1186/1476-069X-9-19

**Published:** 2010-04-26

**Authors:** Anne B Knol, Pauline Slottje, Jeroen P van der Sluijs, Erik Lebret

**Affiliations:** 1 National Institute for Public Health and the Environment (RIVM), Bilthoven, the Netherlands; 2 University of Utrecht, Institute for Risk Assessment Sciences, Utrecht, the Netherlands; 3 Copernicus Institute for Sustainable Development and Innovation; Utrecht University, the Netherlands; 4 Recherches en Economie-Ecologie, Eco-innovation et ingénierie du Développement Soutenable, Université de Versailles, Saint-Quentin-en-Yvelines, France

## Abstract

**Background:**

Environmental health impact assessments often have to deal with substantial uncertainties. Typically, the knowledge-base is limited with incomplete, or inconsistent evidence and missing or ambiguous data. Consulting experts can help to identify and address uncertainties.

**Methods:**

Formal expert elicitation is a structured approach to systematically consult experts on uncertain issues. It is most often used to quantify ranges for poorly known parameters, but may also be useful to further develop qualitative issues such as definitions, assumptions or conceptual (causal) models. A thorough preparation and systematic design and execution of an expert elicitation process may increase the validity of its outcomes and transparency and trustworthiness of its conclusions. Various expert elicitation protocols and methods exist. However, these are often not universally applicable, and need customization to suite the needs of a specific study. In this paper, we set out to develop a widely applicable method for the use of expert elicitation in environmental health impact assessment.

**Results:**

We present a practical yet flexible seven step procedure towards organising expert elicitation in the context of environmental health impact assessment, based on existing protocols. We describe how customization for specific applications is always necessary. In particular, three issues affect the choice of methods for a particular application: the types of uncertainties considered, the intended use of the elicited information, and the available resources. We outline how these three considerations guide choices regarding the design and execution of expert elicitation. We present signposts to sources where the issues are discussed in more depth to give the newcomer the insights needed to make the protocol work. The seven step procedure is illustrated using examples from earlier published elicitations in the field of environmental health research.

**Conclusions:**

We conclude that, despite some known criticism on its validity, formal expert elicitation can support environmental health research in various ways. Its main purpose is to provide a temporary summary of the limited available knowledge, which can serve as a provisional basis for policy until further research has been carried out.

## Background

It is widely recognized that exposure to environmental factors can cause adverse health effects. Many environmental health professionals are confronted with questions about the overall impact of environmental stressors on (public) health, or about the beneficial effects of policy measures to reduce environmental exposures. Oftentimes these questions are extremely difficult to address, due to limitations and inconsistencies in the scientific knowledgebase. Thus, benefits of policy measures directed to reduce environmental exposures are difficult to gauge and policy measures often have to be taken without conclusive scientific evidence. Integrated environmental health impact assessment (IEHIA) aims to support policy making by comprehensively assessing environmental health effects, while taking account of underlying complexities. In this paper, we will use the term IEHIA, but other (similar) forms of impact assessment (e.g. risk analysis, integrated assessment, etc) may serve the same purpose (see e.g. [1]). These types of assessments are often prone to accumulation of uncertainties [1-3].

Formal expert elicitation is one of the means towards a structured and transparent way to address such uncertainties. It refers to a structured approach of consulting experts on a subject where there is insufficient knowledge and seeks to make explicit the published and unpublished knowledge and wisdom of experts. Expert elicitation can serve as a means to synthesize the (limited) available knowledge in order to inform policies which have to be made before conclusive scientific evidence becomes available. The quality of the knowledge derived from experts, or at least its transparency and reproducibility, improves when expert elicitation is applied according to a systematic protocol. Halfway through the last century, the use of such formal expert elicitation arose in disciplines such as systems theory and decision analysis [4]. The Delphi method [5-10] was one of the first formal expert elicitation methods. Over the years, many other methods [7,11-21] and studies have been published. Several (inter)national agencies have made use of expert elicitation, including the IPCC [22], European Environmental Agency [23] and U.S. Environmental Protection Agency [24].

**Figure 1:**
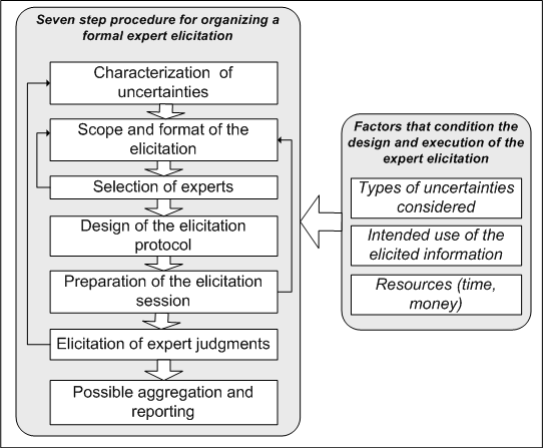
**Seven step procedure for a formal expert elicitation**.

**Figure 2:**
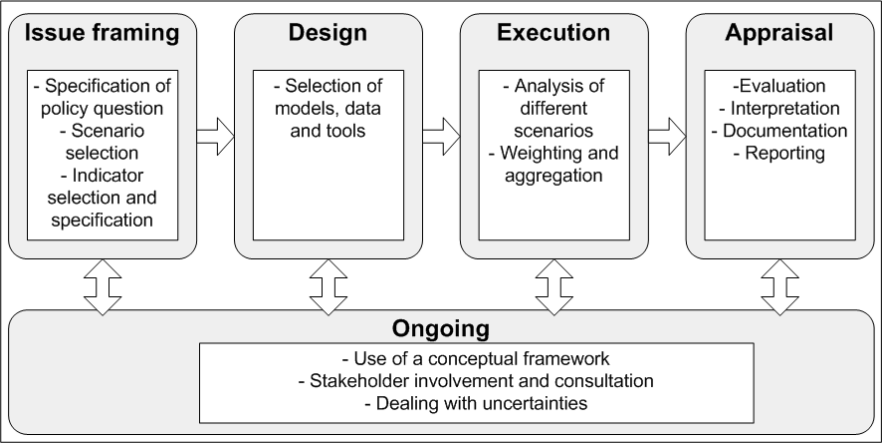
**The phases in the process of IEHIA (based on **[1]). The figure shows the 4 main stages of IEHIA: 1) Issue-framing, in which the assessment boundaries and objectives are specified, 2) Design of the assessment, in which a formal assessment protocol is constructed and the data, models and tools necessary for assessment are specified; 3) Execution, in which the actual (technical) assessment is carried out; and 4) Appraisal, which involves evaluation of the results and their translation into relevant recommendations for action. The activities specified under 'ongoing' take place throughout the assessment, and are of specific importance for the quality of the IEHIA.

As yet, we are not aware of any existing formal expert elicitation protocol that appears flexible enough to deal with the broad range of uncertainties that can be encountered in IEHIA. Therefore, we set out to outline a procedure for formal expert elicitation in IEHIA as guidance for environmental health professionals and users of such assessments, and as part of the EU-funded Intarese project http://www.intarese.org. This paper illustrates the wide potential applicability of expert elicitation in environmental health research and provides practical guidance for its implementation. We do not attempt to describe all available methods for expert elicitation (for reviews the reader is referred to e.g. [25-29]). Instead, this paper focuses on how such methods can be applied in IEHIA, discusses their advantages and drawbacks, and provides references to sources where the issues are discussed in more depth. We specifically focus on the use of expert elicitation in IEHIA that aims to support policy making. This should be distinguished from the use of expert knowledge in e.g. court decisions (expert witnesses), industry or in the media, for which other processes, considerations and expert qualifications are likely to be more appropriate [13,30,31]. We take a broad perspective on the use of expert elicitation in IEHIA: it can be used not only to acquire quantitative figures, but also to gain information about assumptions or causal models. We describe a seven step procedure for organizing formal expert elicitation, which draws from several existing protocols [7,11-21,29,32,33], which consists of the following building blocks: (1) characterisation of uncertainties; (2) scope and format of the elicitation; (3) selection of experts; (4) design of the elicitation protocol; (5) preparation of the elicitation session; (6) elicitation of expert judgments; and (7) possible aggregation and reporting (see Figure 1). We illustrate how the design and execution of these steps is determined by three main issues. First, the type of uncertain information to be elicited; second, the purpose of the elicitation (i.e. how the elicited information is intended to be used) and third, the available resources. The intended use of the elicited information purpose is, among other things, related to the phase of the IEHIA process that the elicited information is to support. The four phases that determine the IEHIA process (issue framing, design, execution and appraisal) are shortly outlined in Figure 2. Briggs [1], amongst others, has argued that more qualitative methods, such as expert elicitation, need to be applied in IEHIA when necessary data or knowledge are lacking, which is often the case in the assessment of complex environmental health risks.

We illustrate the seven step procedure for formal expert elicitation using examples from three existing expert elicitation studies in the field of environmental health research: one on ultrafine particle exposure [34], one on genetically modified crops [35] and one on Campylobacter transmission [36]. The case-studies were chosen because they differ on the three main issues mentioned above and, hence, used different designs.

### Example 1: Exposure to ultrafine particles and potential health effects [34]

Ultrafine particles are a very fine (aerodynamic diameter of <100 nm) component of particulate matter air pollution. They may play a role in initiating or aggravating adverse health effects in humans. As yet, insufficient information is available to assess the magnitude of potential ultrafine particle-related health effects. An expert elicitation was organized to assess the evidence for a causal relationship between exposure to ultrafine particles and health effects and to gain insight into the likelihood of several (patho)physiological mechanisms to explain a potential causal relationship. The resources available for this elicitation were sufficient. The study has been planned and organized using the seven step procedure for expert elicitation as presented in [37], which also form the basis of this manuscript.

### Example 2: Genetically modified herbicide-resistant crops and potential adverse effects on agricultural and cultivation practices [35]

By genetic modifying plants, it is possible to develop crops that are able to break down a particular herbicide, which makes the crops resistant to that herbicide. As such, weed control can be made simpler, as herbicides can be applied without damaging the crops themselves. However, such genetic modifications might have partly unknown and potentially adverse effects on agricultural and cultivation practices, and potentially also on public health. An expert elicitation was organized in order to obtain qualitative and quantitative information regarding the uncertainties present in agricultural risk assessments on GM oilseed crops (canola or rapeseed). No formal protocol was used during this elicitation, as none existed for the broad concept of uncertainty employed here. The available resources were limited.

### Example 3: Broiler-chicken processing and potential exposure to Campylobacter [36]

When broiler-chicken carcasses are processed, there is a risk that pathogens such as Campylobacter are transmitted. When the broiler-chicken meat is consumed, this may lead to Campylobacter exposure in the population, posing a serious health threat. A formal expert elicitation study was organized to quantify model parameters for a risk assessment model describing the transmission of Campylobacter during broiler-chicken processing in the Netherlands. The model aims to assess exposure of the Dutch population to Campylobacter as a consequence of the consumption of broiler-chicken meat, as well as to compare the effects of various intervention strategies to reduce this exposure. Experimental data on the model coefficients were not available, and experts did not have enough information to reasonably quantify these values and their uncertainties. Instead, they were asked to provide probability distributions for other quantities with which they were sufficiently familiar to render a judgment. From these estimates, the necessary model parameters could be derived using probabilistic inversion techniques [38]. The organizers have used a protocol for expert elicitation developed by Cooke and Goossens [11] as a basis for designing their study. The resources available for this elicitation were sufficient, as it was part of a larger project in which it was budgeted for (Arie Havelaar (AH), personal communication, March 2009).

## Seven step procedure for formal expert elicitation

Figure 1 shows a seven step procedure for formal expert elicitation. Similar steps are recognised in most existing protocols [7,11-20,29,32,33], which have formed the basis for this protocol. In practice, the elicitation procedure will often not be strictly chronological, but instead an iterative process with feedback loops. Below, the seven steps are described and illustrated with examples from the expert elicitation studies that are introduced above.

### Step 1: Characterisation of uncertainties

Expert elicitation is one of several methods to deal with uncertain information in IEHIA. Other methods include for example modelling missing data, scenario analysis or sensitivity analysis [21]. The characterisation of uncertainty determines whether expert elicitation is a relevant approach to deal with the uncertainties in a particular IEHIA. Uncertainties can, for example, be quantitative or qualitative; reducible or permanent; dependent on different measurement methods or on different personal values held by the scientists. An uncertainty typology [3,39-43] may help to identify and characterise the different types of uncertainties in a specific study, and point to methods to deal with these uncertainties. An example of such a typology is presented in the following paragraph, illustrated with examples from expert elicitation studies related to environmental health. Van der Sluijs et al. [43], using a similar uncertainty typology as the one we present below, have described a range of methods to deal with uncertainties (including expert elicitation). They provide recommendations about which methods are most appropriate, according to the characteristics of the specific uncertainty. For example, sensitivity analysis can be useful for dealing with statistical uncertainty, but is less suitable for recognized ignorance (see for an explanation of these terms the following paragraph). Expert elicitation is recognized as a widely applicable method [43]: it can be used to identify and reduce many different types of uncertainties.

The typology described here is useful in the scientific analysis of uncertain environmental health issues. When the results of such analyses are communicated to policy makers or other stakeholders, further uncertainty can derive from vagueness or ambiguity in the wording and presentation of the results. This type of uncertainty, referred to as linguistic uncertainty [44] or linguistic imprecision [14], is further discussed in the paragraph on the design of the elicitation protocol.

#### Typology of uncertainty

The typology of uncertainty, which we have presented more extensively earlier [3], distinguishes the following dimensions: 1) the location of uncertainty, 2) its nature, 3) its range, 4) its level of recognized ignorance, 5) its level of methodological unreliability, and 6) its level of value diversity among analysts. Each piece of uncertain information can be characterized along each of these dimensions. We will illustrate the typology of uncertainties with examples from expert elicitations in the field of environmental health research.

1) The location of uncertainty specifies where in an IEHIA uncertainty manifests itself. Different locations can be distinguished: the context of an assessment, the underlying model, the parameters and the input data.

The context of an IEHIA relates to the definitions and boundaries of the assessment: what are we assessing? This question is addressed in the 'issue framing' phase of an assessment (see Figure 2). Although expert elicitation is not (yet) commonly used for defining boundaries or scenarios, some examples exist. In the GM-crop elicitation, Krayer von Krauss et al. [35] have used expert elicitation to gain insight into potential issues of concern that might currently be ignored in assessments on the risks of genetically modified crops. Similarly, Beerbohm [45] has elicited expert views about the biggest risks of re-introduction of DDT for managing malaria. As such, they gained insight into the greatest risks as perceived by experts.

Model structure uncertainty relates to incomplete understanding of the ways in which environmental factors are causally related to health. Conceptual (causal) models may be used to represent this structure graphically. They are primarily designed in the 'issue framing' phase of the assessment and further specified in the 'design' and 'execution' phase (Figure 2) (Knol et al., in press). In order to address model structure uncertainty, experts may be asked to (a) construct a conceptual model; (b) judge the likelihood of several proposed models, or (c) provide knowledge on a particular subject, which can subsequently be used to form conceptual models. An example of the first approach is provided by Evans et al. [46], who asked experts to construct a probability tree for cancer causation. The second approach was pursued by Havelaar et al. [47], who used expert elicitation to judge the fraction of enterically transmitted illnesses that could be explained by alternative pathways. Similarly, experts participating in the ultrafine particle elicitation [34] were asked to judge the likelihood of several proposed patho-physiological pathways. The third approach has been employed by Hoffman [48], who asked experts, amongst other things, which types of food they would (qualitatively) exclude from being able to transmit a certain agent. As such, this study contributed to better understanding of the underlying causal pathways to foodborne illnesses.

Parameter uncertainty describes uncertainty of parameters used in an assessment, such as those used in exposure-response functions. Parameters, as well as input data (see next), are used in statistical analysis of a model in the 'execution' phase of IEHIA (Figure 2). Uncertainties of parameters are usually well considered. Exposure-response functions are, for example, typically presented with confidence intervals. Parameters are also the most common subject of expert elicitations, and many examples in the environmental health literature exist. In the Campylobacter-elicitation [36], experts were asked to estimate several in- and output variables of a broiler-chicken processing model. From these estimates, the actual model parameters could be derived using probabilistic inversion techniques [38]. As another example, disability weights, which are used in summary health impact measures (e.g. environmental burden of disease estimates such as DALYs) to define the severity of a disease, are by definition based on expert judgments [49]. Also in air pollution research, expert elicitation has been used on various occasions to estimate parameters of exposure-response functions (e.g. [50,51]).

Input data uncertainty describes uncertainty in datasets used in the 'execution' phase of IEHIA, such as exposure data or disease incidence data. Expert elicitation can be used to estimate the uncertainty of existing datasets. Walker et al. have used expert elicitation to obtain distributions of benzene concentrations [52]. Similarly, Oorschot et al. [53] have elicited information about uncertainty in NO_x _emission data.

2) The nature of uncertainty characterizes whether the uncertainty is primarily caused by incomplete knowledge (epistemic uncertainty) or by intrinsic properties of the system itself (ontic uncertainty). The latter refers to natural variability of a system, such as weather conditions or activity patterns. Most of the examples mentioned so far are examples of epistemic uncertainty: there is a lack of knowledge and expert elicitation is used to fill the gap. However, ontic uncertainty can also be the subject of an expert elicitation, if its extent is poorly known. For example, Titus and Narayanan [54] have estimated both epistemic and ontic uncertainty of parameters in a model estimating the probability of future sea level rise from climate change. Similarly, Oorschot et al. [53], in their elicitation on uncertainty in NO_x _emission data, addressed both epistemic and ontic uncertainty. The latter, referred to as aleatory uncertainty in their report, was for example found in variability of numbers of cars.

3) The range of uncertainty gives an indication about whether an uncertainty can be expressed in statistical terms (i.e. as a subjective probability distribution), or in terms of scenarios (i.e. as a range of plausible events, without any definitive information about the relative likelihood of each scenario). This range of uncertainty affects the format of information to be elicited, e.g. as subjective probability density functions or as relative likelihoods of scenarios. The examples about parameter uncertainty [36,50,51] mentioned above mostly involve statistical uncertainty: these parameters could be adequately expressed in statistical terms. In the examples provided for context uncertainty [35,45] and model structure uncertainty [46-48], no such statistical estimates were warranted and experts were instead often asked to judge the likelihood of various scenarios using a qualitative likelihood scale.

4) Recognized ignorance deals with aspects of uncertainty for which we cannot establish any meaningful estimate. In such cases, expert elicitation can be used to give insight into what is not known and to what extent this is considered important. Krayer von Krauss [35] identified two areas where knowledge was considered insufficient in genetically modified crop research. Hawkins et al. [55] made experts judge the validity of extrapolating tumour data observed at high carcinogen exposures to lower exposure levels. These lower levels were below the experimental range, so the assumptions about extrapolation could not be validated: a case of recognised ignorance.

5) Methodological uncertainty reflects weaknesses in methodological quality of (part of) an assessment. This may relate to its theoretical foundation, empirical basis, reproducibility or acceptance within the peer community. Expert elicitation can be used to identify or prioritize areas of potential methodological uncertainty. Jelovsek et al. [56] have used expert elicitation to gain insight into the rules-of-thumb that are used to determine if a compound or agent is likely to be a developmental hazard during pregnancy, and judged the consensus among experts about these principles.

6) The final dimension of uncertainty distinguished here is value diversity among analysts, which relates to personal values and normative judgments held by scientists (and not to numerical values). Value diversity occurs when different, potentially valid choices can be made about assumptions in an assessment. The assessors making these choices may have different normative values and hence make different choices [19]. The expert elicitations mentioned above about deriving severity weights for diseases [49]; the validity of extrapolating tumour data [55]; and the principles used to judge hazardousness of agents during pregnancies [56] all have a degree of value diversity: expert opinions are likely to vary on these issues because experts may rely on different personal norms and beliefs.

### Step 2: Scope and format of the elicitation

Resources (time and money) often limit the scope of an expert elicitation: how many experts can be approached; can experts be compensated for time and additional expenses; can international experts be invited, etc [57]. In general, if the information to be elicited is critical for policy making, when the outcomes are likely to be used in delicate (court) decisions, or if value diversity is high, a more elaborate expert elicitation is warranted. In contrast, one might opt for a more confined approach when new relevant information is foreseen in the near future, or when the body of evidence is relatively extensive and consistent.

#### How many experts?

There is no absolute guideline on which to base the number of experts to be invited. According to a panel of expert elicitation practitioners [57], at least six experts should be included; otherwise there may be questions about the robustness of the results. The feeling of the practitioners was that beyond 12 experts, the benefit of including additional experts begins to drop off.

#### Group or individual elicitation?

Resource-wise, personal interviews generally consume more time of those organising the expert elicitation, but less money, as compared to group elicitation sessions. The potential benefits of group interaction include sharing of knowledge and better appreciation of different disciplinary viewpoints [58]. On the other hand, individual interviews may allow for more targeted questions and explanation. Downsides of group interaction include inappropriate dominancy of 'influential experts' and the implicit suggestion of the 'need to achieve consensus'. Even though in the traditional Delphi method consensus was indeed sought for, disagreement among experts may in fact indicate important information [13], and looking for consensus is not always appropriate.

#### Interviews or surveys?

Information can be elicited from experts in various ways: by conducting interviews, by having questionnaires filled out, or by using specific software [7,59-64]. In general, face-to-face interviews are preferable. This leaves more room for explanation, experts might be more motivated to join, and they may feel more responsible for providing informed judgements to an interviewer or a group than to an anonymous questionnaire. On the other hand, internet or postal questionnaires are less expensive, their content can be better standardized than the content of personal interviews, and experts may complete them at their leisure.

In the ultrafine particle elicitation [34], experts from various disciplines were invited in order to stimulate interdisciplinary discussion. The elicitation described here was combined with another elicitation on ultrafine particles on the next day [65], in order to use resources efficiently. The total budget for the two UFP elicitation days was around 20,000 euros. This estimate includes organisation and accommodation and transport for the experts, but excludes any time spent on preparation, analysis and reporting. The budget allowed for a two day session with 12 experts. In order to reduce travel costs, only experts based in Europe were selected. The total budget of the Campylobacter-elicitation [36] was estimated to lie roughly around 50,000 euros (AH), including time spent on analyses and reporting. The team interviewed 12 experts individually. This approach was chosen in order to explain the process to each expert step-by-step. It was considered that experts would be more motivated to cooperate if their opinions were apparently considered important enough for a personal interview (AH). A similar motivation made the organizers of the GM-crop elicitation [35] decide to conduct individual interviews. Moreover, as no specific funding was available for the GM-study (it was carried out for minimal costs by a PhD student), it was not possible to organize a group meeting, since experts could not be reimbursed for travel or accommodation costs. The investigators identify the possibility that different experts might have had different understandings of the questions [35]. A group workshop might have prevented this to some extent. Indeed, this was recognised as one of the main benefits of group elicitation recognized in the ultrafine particle elicitation.

### Step 3: Selection of experts

#### Types of experts

Which experts take part in an elicitation can greatly affect its outcomes and their acceptability in the wider community, so the selection of experts requires careful consideration [10,13,66]. The experts we refer to in this manuscripts are professionals (scientists, technicians, physicians, etc.). However, it is increasingly recognized that non-professionals (for instance a patient or a government official) can also contribute valuable information and perform well in the elicitation of subjective opinion. For that kind of involvement, other participatory methods than the type of expert elicitation we describe here may be more suitable (see e.g. [67,68]).

In general, three types of professional experts can be distinguished: generalists, subject-matter experts, and normative experts [13,17]. Generalists typically have substantial knowledge in a relevant discipline and a solid understanding of the context of the problem. They can be particularly useful in expert elicitations about context or model structure uncertainties (see 'Typology of uncertainty') and are useful when the topic is multidisciplinary. Subject-matter experts are typically regarded by their peers as an authority in their field of expertise. They are the prime experts from whom judgements are often elicited, and they are essential for estimating subject-specific information, such as model parameters. Normative experts, finally, have knowledge, practical experience or skills that can support the elicitation process itself. They may for example be specialized in decision analysis, statistics or psychology. These experts can assist when thought processes are challenging or when the format of the elicited information requires insight into probabilities or heuristics.

In the ultrafine particle elicitation [34], experts were selected from three different disciplines: epidemiology, toxicology and clinical medicine. Primarily subject-matter experts were invited, of which some could function as generalists because they were aware of developments in other fields. A normative expert was member of the organizing team. In the GM-crop and Campylobacter-elicitations [35,36], only subject-matter experts were selected as in-depth knowledge on the subject was needed in order to make informed judgements.

#### Balance

When there is a high degree of value diversity (see 'Typology of uncertainty'), when there are high stakes involved, or when results need to be accepted by a wide peer-community, it is particularly important to have a well-balanced expert panel. Opposing views need to be justly represented in the panel and experts should preferably not have strong commitments to a particular outcome. In order to enhance such balance, it is recommendable to use a formal selection procedure [13,15], as was for example applied in the ultrafine particle expert elicitation [34]. There, the organisers applied a two-step nomination procedure. First, they selected authors of at least two peer-reviewed papers on the subject of ultrafine particles and health effects using a systematic literature review. These people were asked to nominate experts who had, in their opinion, the necessary educational background and/or experience to participate in the expert elicitation. The scientists who got most nominations were subsequently invited. In the GM-crop elicitation [35], some effort was made to include different perspectives, by interviewing experts from both government and industry. It proved to be difficult to involve people from regulatory authorities, both because they were too busy to make themselves available, and because they felt uncomfortable critically reviewing a prior risk assessment carried out by another regulatory agency (Martin Krayer von Krauss (MKvK) personal communication, March/April 2009) The elicitation on Campylobacter transmission [36] actively sought for experts from different disciplines. They included the restriction that only one expert per organisation could participate. The latter restriction was also employed in the ultrafine particle elicitation.

#### Availability of expertise

The elicitation of information from experts hinges on the availability of expertise in the scientific community. Experts cannot make up knowledge that does not exist yet in one form or another. However, when issues are highly uncertain, controversial, unquantifiable or associated with potentially irreversible damage; or when decision stakes are very high [69,70], there may be insufficient expertise available to derive any valid judgments. Further training in, for example, providing subjective probability distributions, or more extensive discussions between experts, will not compensate for such unavailability of expertise. For these types of issues, expert elicitation cannot be a panacea.

### Step 4: Design of the elicitation protocol

#### Types of information to be elicited

The elicitation protocol contains the questions to be asked during the elicitation and the desired format for the answers. Expert elicitation can be used both for quantitative and qualitative estimates, as well as for the construction or evaluation of conceptual (causal) models.

Quantitative estimates are often elicited for parameter and input data uncertainty (see 'Typology of uncertainty'). These values are necessary in the execution phase of the assessment, when the relevant models and analyses are run. Estimates are often expressed in probabilistic terms (min, max and most likely values; subjective probability density functions), such as used in the Campylobacter-elicitation [36]. Experts can be asked to provide such estimates directly. Alternatively, values can be derived indirectly, for example by asking related questions from which the values can be derived [64]; by having experts provide a graphical representation of e.g. an exposure response curve; or by letting experts view and alter spatial data in a geographical information system (as applied in [63]).

Commonly, quantitative information is elicited in the form of a number, its unit (e.g. grams or euros) and its uncertainty. The NUSAP approach [16] has been developed in order to complement this standard information with a qualitative assessment of the part of uncertainty that cannot be captured in a number. NUSAP stands for Numeral, Unit, Spread, Assessment and Pedigree. The Assessment provides a qualitative judgement about the reliability of the estimate. The Pedigree conveys the way in which the estimate was produced and provides insight into its strength. NUSAP provides a means to assess and communicate uncertainties (input data, parameter as well as certain forms of context and model structure uncertainty) in a harmonized way, based on both quantitative and qualitative information about the underlying knowledge base [16]. As such, it provides valuable interpretative information about individual expert judgements, and helps to pinpoint the locations and severity of any disagreements between experts.

Context and model structure uncertainty (see 'Typology of uncertainty') can usually only be addressed in a qualitative way: to determine which data and variables are relevant for analysis; to judge which analytical methods are appropriate; or to assess which assumptions are valid, as elicited in the GM-elicitation [35]. Such qualitative information is needed in the issue framing and design phase of assessment to define the conditions on which the assessment is based. Sorensen et al. have for example explored the use of mind mapping tools to define system boundaries (context uncertainty) in the assessment of chemicals and nanomaterials, and as such elucidated sources of recognized ignorance in these assessments [71].

Conceptual models can be used to graphically represent the causal relationships between different variables. The elicitation of conceptual models (model structure uncertainty) can be aided by using group model building techniques [72]. These help people to learn from each other and build a shared perspective. The format of questions depends on the degree of freedom given to the experts: should they construct a model from scratch, position a set of existing variables and draw the linkages between them, or adapt or evaluate existing model(s)? Zio and Apostolakis [73] have compared two approaches for quantitatively assessing model structure uncertainty using expert elicitation. First, they have explored the alternative-hypotheses approach, in which experts are asked to express their beliefs about the probability (likelihood) of several plausible models. This method is very similar to the one applied in the ultrafine particle elicitation, although in that elicitation no overall estimate of model structure uncertainty was elicited quantitatively [34]. Second, they discuss the adjustment-factor approach, in which a 'best' model is identified and an adjustment factor is used to represent model uncertainty. They conclude that the latter approach is theoretically less defensible, but may be more practical and flexible. Which of these two methods is more appropriate for a specific question is context-specific [73]. Finally, the format of the elicitation questions naturally depends on the purpose of the conceptual model: is it meant to outline the broad general structure of the assessment at hand as input for the issue framing or design phase, or should it portray exact causal relationships between variables as a basis for analysis in the execution phase of assessment? A clear explanation of the type of conceptual model required and the rules on which the development of the model should be based supports model development by experts.

#### Performance and (internal) consistency of experts

Some experts may be better at making valid judgements than others. However, it is often difficult to check such performance of the experts, as the 'true values' of their estimates are unknown. Therefore, so-called seed variables can be included. The actual (measured) values of these seed variables are unknown to the experts, but known to the analysts. The performance of the experts on assessing these variables can be used as a proxy for their performance on the query variables, which are the variables of actual interest. Seed variables were for instance included in the Campylobacter-elicitation [36] (see also paragraph on aggregation of results below).

Another way to assess performance, or at least internal consistency, is to have experts judge one outcome in two or more different ways [74]. This method has been employed in the GM-elicitation [35], in which experts were asked to rank the relative sensitivity of the conclusion of the risk assessment to each of the specific sources of uncertainty. In this case, the experts were first asked to prioritize the list of uncertainties on a 0-1 scale, with 0 meaning 'large variation has small effect on assessment conclusion' and 1 meaning 'small variation has large effect on assessment conclusion'. Second, they were asked to allocate poker chips to uncertainty sources which indicated how much they would be willing to invest to completely eliminate that uncertainty. For internally consistent experts, these two approaches should lead to (roughly) the same priority list of important sources of uncertainty. In this specific elicitation, consistency was not very high. This could either be explained by incomplete understanding of the questions, or by the use of different underlying motives for the two different assignments. Further exploration of underlying reasons for inconsistency was not feasible in this specific study, but is in our view recommendable.

#### Wording of questions

The wording and phrasing of questions needs careful consideration, as it may substantially affect the given responses [29,75]. Even slight rephrasing of the same question has been shown to lead to differences in (quantitative) responses of 4 to 15% [29]. Such linguistic uncertainty [44] can be classified into four main types: vagueness, context dependence, ambiguity, and underspecificity. Vagueness (e.g. the meaning of "likely") can be addressed by providing clear definitions, and, when possible, numerical orderings that relate to the vague statements. Context dependence of questions calls for provision of sufficient background information about the context for which the statements are to be valid. For example, in the ultrafine particle elicitation [34], experts were explicitly told to assume that a causal relationship between exposure to ultrafine particles and cardiac events existed while make statements about the likelihood of specific causal mechanisms. Linguistic ambiguity arises when words can have two meanings and it is unclear which meaning is meant. Finally, underspecificity occurs when too much room for interpretation is left because not enough details are provided. For example, in the Campylobacter elicitation [36], many details about the broiler-chicken processing were provided in order for the experts to condition their estimates in the same way.

Elicitation questions are thus best formulated in a manner which is consistent with the way the experts commonly present their knowledge [11], presented with sufficient background information and open for one interpretation only [14]. The exact wording of the questions might be open for discussion during the elicitation session. This may increase common understanding and approval of the questions and is particularly appropriate in an interdisciplinary expert elicitation, because semantics may differ between disciplines. Changing the questions is not recommendable in a series of individual elicitation sessions (such as personal interviews), as this diminishes the inter-expert comparability. In the ultrafine particle elicitation [34], experts were allowed to discuss and slightly alter the wording of the questions, which proved to be highly useful to create common understanding, and reduce the semantic differences between disciplines. Some further guidelines for question phrasing are provided by [11,14,29,75].

Questions can be also pre-tested in order to evaluate their clarity and completeness, which can be done within the organizing team or, preferably, by some people outside this team. In the Campylobacter-elicitation [36], two experts, who did not participate in the actual elicitation, were asked to join a dry-run of the elicitation and comment on the proposed structure of the session. More characteristics of the way answers and responses are framed, ordered and explained, and the ways in which this may affect how they are perceived and answered to, are described in e.g. [76,77].

#### Motivation for answers

It is highly recommendable to ask experts to provide (written) motivations for their judgments and identify issues that affected them. This reduces the chance of heuristics and biases to remain unrecognised, and increases the proper interpretation of final results and potential outliers (see below). All three illustrative elicitations have encouraged experts to provide such supporting information.

**Biases and heuristics **[12-14,26,78-81]

People use various heuristics when judging uncertain information [14]. Some of these may introduce bias in the outcome. Heuristics may for example relate to availability, representativeness, anchoring or adjustment. Availability bias arises if the expert is affected by the ease of recall or the memory of recent experience. The representativeness bias refers to inappropriate generalisation of specific knowledge, or to paying too much attention to specific details at the cost of background information. Anchoring and adjustment relate to the procedure of experts to first select a starting point (an anchor) as a first approximation of the quantity at hand, and then to adjust this value to reflect supplementary information. Results are then typically biased towards the anchor. This may explain part of the frequently observed overconfidence of experts [12], i.e. assigning probabilities that are more certain than is warranted. Overconfidence can become clear when an estimate of a quantity and its uncertainty are given, and it is retrospectively discovered that the true value of the quantity lies outside the interval. Klayman et al. [78]have demonstrated that, in general, there is little overconfidence with two-choice questions and more substantial overconfidence when experts are asked to provide subjective confidence intervals. Also, they have shown that some individuals are more prone to overconfidence than others, and that the topic about which questions are asked affects the general level of overconfidence (independent from the difficulty of the questions). Overconfidence is difficult to guard against, but a general awareness of the tendency is important. It may be reduced by using structured questions and frequency formats [79]. Asking experts to construct careful arguments in support of their judgements may also improve the quality of assessments. In addition, overconfidence may be reduced by asking the experts to list one reason against their choice of response, or by stimulating them to think about extreme high or low values before asking about central estimates. Overconfidence may become apparent when experts are asked to estimate values and confidence intervals outside their field of expertise (e.g. weight of the earth; number of existing insect species). However, it is unclear if overconfidence in relation to such questions is a relevant proxy for overconfidence within the expert's field of expertise.

Bias in response may also results from motivational bias. This occurs when the response of an expert is influenced by factors such as moral or professional responsibility, legal liability or peer credibility. For topics with a high level of value diversity, experts can be particularly prone to motivational bias, because they may want to influence the outcome of the elicitation [80]. This might be partly reduced by asking for careful argumentation for each given judgment. In addition, specific tests (e.g. [81]) can be used to identify the implicit values and attitudes held by researchers, which could lead to motivational biases. Hindsight bias, finally, refers to the tendency of people to exaggerate the predictability of reported outcomes, apparently because they fail to think about how things could have turned out differently.

### Step 5: Preparation of the elicitation session

Experts can be provided prior to the elicitation with the program for the expert elicitation and the protocol with the questions to be posed (see previous paragraph). In addition, background information about the IEHIA and the subject of the elicitation can be provided to the experts in a so-called briefing document. The information provided in this briefing document should balance potential disparate and disciplinary views (especially when a high level of value diversity exists), and may contain any of the following elements [11,12]:

- Outline of the nature of the problem and the uncertainties related to it, including the conditions on which the information is to be elicited;

- Key literature; optionally inviting experts to add missing papers;

- A (qualitative or quantitative) summary of the literature. This might however unintentionally stimulate the experts to use primarily the provided material in their judgment;

- Information about the elicitation procedure;

- Information about heuristics and biases (see above section).

The organizers of the ultrafine particle expert workshop [34] provided all these elements in their briefing document. In the Campylobacter-elicitation [36], a special group training session was held prior to the interviews in order to discuss the documentation and to train the experts in estimating probabilities (see next paragraph). Technical details on the model and information on probabilistic thinking were provided in the interviews, adapted to the needs of the individual experts (AH). In the GM-elicitation, the purpose, case-study details, and information on uncertainties were reviewed with the experts at the start of each interview. This approach was adopted in order not to overwhelm the experts with complex information prior to the interview. It was assumed that experts would probably not read or understand all of it completely, and therefore it appeared more reliable to explain everything personally (MKvK).

### Step 6: Elicitation of expert judgements

#### Introduction of the scope and purpose of the expert elicitation

At the start of an expert elicitation session, typically an introduction will be held about the field of interest and the purpose of the meeting in order to familiarize the experts with the subject matter. During this introduction, the uncertainties at hand and the elicitation format can be discussed. Experts can be informed about what is expected from them and how results will be used and distributed. It is important for this purpose to be communicated clearly to the experts and adhered to in the follow-up of the elicitation. Experts may, for example, be willing to make judgements if these are to be used for purely academic purposes, but not if they are meant to form the basis for policy regulations or court decisions.

#### Pre-elicitation training

If quantitative estimates are to be made, the use of training questions is advised, because most experts are unfamiliar with quantifying their degree of belief in terms of probabilities. These test questions can also be used to explain and discuss the format of the elicitation [17]. Experts may furthermore need to be made aware of potential heuristics and biases. In the ultrafine particle elicitation [34], a normative expert started the meeting with a presentation about biases and heuristics. As mentioned earlier, the Campylobacter-elicitation devoted a separate training session to these types of issues [36].

#### Elicitation of judgments

Means to guide experts in correctly synthesising their judgements in an expert elicitation have been described in previous sections. Possible explanations for differences in judgments between experts include [16] (a) different background information on which the experts base their judgement; (b) different interpretation of the linguistic descriptions; and (c) disagreement among experts on a more fundamental level. The first two causes (especially the second) need to be avoided. They can be minimized for example by a group discussion prior to the elicitation to come to a shared understanding of the questions. Also, a dry-run prior to the elicitation with experts other than those joining the elicitation, as employed by the Campylobacter-elicitation [36], can reduce these unwanted causes of variation. When individual interviews are carried out, it is furthermore recommendable to keep at least one member of the interview teams similar in order to minimize potential differences. In the Campylobacter-elicitation [36], interviews were held by three different elicitation teams. They had been given instructions beforehand in order to minimize differences in approach.

#### Post-elicitation feedback

Post-elicitation feedback can be given instantaneously (as in computer-assisted elicitation) or delayed (e.g. on paper) and may serve multiple aims. First, it enables experts to check whether their results reflect their thoughts, and revise if necessary. Second, it stimulates discussion, as individual results can be shown in relation to the judgements of others, in order to identify interpretation differences and potential mistakes. However, this might (un)consciously stimulate experts with extreme ratings to move towards what most others reported, which could result in unwanted regression to the mean.

The elicitation on ultrafine particles consisted of a round in which experts individually gave a first estimate, followed by a group discussion and then a final individual rating. Any changes in ratings between rounds were motivated by the experts. These changes were considered to be mostly an effect of contemplating new arguments or of a more harmonized interpretation of the question, rather than of 'peer pressure' or anchoring [34]. However, peer pressure is rather common, difficult to identify and difficult to completely reduce, so it is well possible that some anchoring effect has remained. The assessments made by the experts in the Campylobacter elicitation [36] were returned to them after the elicitation in order for the experts to confirm their results. No information was given on the individual performance of the experts on the seed variables (AH). Finally, in the GM-elicitation [35], experts were sent the manuscript for review. No further comments were received (MKvK). Experts participating in the ultrafine particle elicitation were invited to co-author the manuscript, which all but one expert chose to do.

### Step 7: Possible aggregation and reporting

#### Aggregation of results

Expert judgments can be summarized into one single estimate, or they can be presented individually. There is no consensus among scientists about the conditions under which aggregation is warranted and if so, in what way [58,82,83]. In general, diversity of expert views itself carries valuable information and should be part of the open reporting of the study results. The fraction of experts who give a particular estimate might not be proportional to the probability of that estimate being correct [82]. Therefore, combining judgments might become problematic. Especially when a high level of value diversity exists, it might be more appropriate to report disparate views. However, some form of aggregation may sometimes be necessary in order to facilitate use and comparison of results.

If quantitative estimates such as probability density functions are to be combined into one final estimate, assessors have the choice of various methods to weigh the individual estimates of the experts. One of the most obvious is the equal weighting scheme, in which the estimate of each expert is counted equally in the summary estimate. However, various more complex aggregation processes can be applied [25,58,84], which usually involve valuing the judgments of some experts more than those of others, based on an estimate of their performance. This can be derived from the quality of the estimates that experts made on the seed variables, if a sufficient number of good quality seed variables -generally about 8 to 10- are available [36]). Aggregate quantitative values were needed in the Campylobacter-elicitation [36] in order for the parameters to be applied in the final model. The elicited values were weighted using three different weighting schemes, using specific software [59]. The weights used were based on the experts' performance on the 12 identified seed variables. All three weighting schemes had adequate performance, but one specific scheme (using optimized combined distributions) provided significantly better results and was used for further processing the results. The estimate of performance of the weighting schemes was based on two measures: calibration (the statistical likelihood that the experts assessments correspond to the actual measured values of the seed variables) and information (related to the distribution and uncertainty of the experts assessments) [84].

As an alternative to performance based on seed variables assessments, experts may be qualified according to some other consistency test (see section on format of questions); their number of scientific publications or peer-nominations [85]; or according to their own subjective judgment of their "level of certainty" about a particular elicited value [11]. However, it is doubtful whether such estimates provide a good indication of the actual value of the elicited information, or merely introduce more bias [11,85].

Instead of statistical aggregation, one might also attempt to derive a summary estimate by generating agreement among the experts [58] (cf. the original Delphi method [8,9]). Both mathematical as well as discussion-based approaches tend to be similar in performance [58]. This raises the question whether sophisticated aggregation techniques provide any added value over a simple group discussion. This is especially questionable when experts have shown difficulty in expressing their beliefs in probability density functions, due to the complexity of the topic or their unfamiliarity with the process of elicitation. Ways to measure the quality of expert judgments are scarcely available (besides methods described earlier such as the use of seed variables), and often controversial. If aggregation of results is desired, the best solution might lie in combining aspects from both mathematical and discussion-based methods for summarizing and aggregating quantitative results [58]. For reconciling qualitative estimates or differences in conceptual models, deriving consensus through discussion is effectively the only option available. Alternatively, scenario analyses can be carried out to compare different conceptual models or qualitative assumptions.

#### Reporting judgments

Proper reporting and presentation of the procedure and results of an expert elicitation is very important [13]. The aim of the elicitation and the anticipated use of the results need to be made clear, in order to prevent that the experts' estimates are used in a context which they were not intended for. It is beyond the scope of this paper to describe the communication of uncertain results to policy makers and other stakeholders, but useful suggestions are provided by e.g. [44,86-89]. We will shortly describe the scientific reporting of the results. In the publication on ultrafine particles [34], the frequency of likelihood ratings are presented graphically and variations in ratings are discussed. In the GM-crop elicitation [35], responses are mostly reported as mean, min and max values, in order to facilitate comparison among topics. However, in subsequent publications [90,91], the authors reported disaggregated results in order to increase transparency and better illustrate the level of disagreement amongst the participating experts, as they considered this to be one of their most important findings. The results of the Campylobacter-elicitation [36] are presented as probability density functions for both seed and query variables.

Individual expert judgements are often reported anonymously, so that experts feel they can respond freely and to avoid some of the possible motivational biases. Specific judgements can be referred to by an arbitrarily assigned number, as has been done in the GM and Campylobacter-elicitations [35,36]. A list of all participating experts can be provided in an acknowledgement statement (as in the GM-elicitation [35]), or experts can be asked to co-author the paper (as in the ultrafine particle elicitation [34]). In the Campylobacter-elicitation, it was announced prior to the interviews that names of experts were not to be mentioned anywhere in the manuscript. This approach was chosen to make experts feel more comfortable giving estimates that could collide with, for example, ideas of other experts or their own previous beliefs (AH). Such complete anonymity can be sensible when a high level of value diversity on the subject exists.

In addition to the expert judgments, the selection and elicitation processes need to be documented, in order to allow for reproducibility and to improve the interpretation of the results. All three studies described here provided sufficient detail about the operational issues involved in the organization of their elicitations, which is of crucial importance for the interpretation of the process and its results.

## Discussion

Expert elicitation can be a useful means to gain insight into environmental health issues about which current evidence is limited or inconclusive. It provides a temporary summary of the limited available knowledge. As such, it can be used as a relatively quick and inexpensive, albeit lower quality, substitute for time or money consuming research, such as long term monitoring projects or cohort studies. The resulting estimates can serve as a basis for action in cases where problems are too urgent or stakes are too high to postpone measures until more complete knowledge is available. Although expert elicitation is most commonly used to estimate quantitative values, it can also provide insight into qualitative issues or conceptual (causal) models. Expert elicitation may help to structure a problem and can be used to focus new research on the most salient uncertain issues. As such, expert elicitation can be applied widely in environmental health research, and provide useful contributions relevant to all four phases of IEHIA. The transparency and reproducibility, and most likely also the quality of the elicited information increases when the expert elicitation is carried out according to a systematic protocol.

In this paper, we have outlined a seven step procedure that can be applied to organise a formal expert elicitation in the context of environmental health research, and IEHIA in particular. It is based upon a broad definition of uncertainty and hence widely applicable. The procedure consists of the following seven steps: (1) characterisation of uncertainties, in which the type of uncertain information to be elicited is identified and characterized according to a typology of uncertainties; (2) scope and format of the elicitation, in which the number of experts to be invited and the most appropriate form of elicitation is determined; (3) selection of experts, in which the necessary types of experts and the balance between different disciplines or viewpoints is considered, preferably using a formal selection procedure; (4) design of the elicitation protocol, in which the types of questions and the appropriate format and wording of the questions and answers are determined, taking into account potential effects of heuristics and biases; (5) preparation of the elicitation session, in which a protocol for the elicitation session is developed and background information is distributed; (6) elicitation of expert judgments, in which the different phases of the actual elicitation (introduction, pre-elicitation training, elicitation of judgements, and post-elicitation feedback) take place; and (7) possible aggregation and reporting, in which the expert judgements may be aggregated and the results of the elicitation are reported. Two recent expert elicitations [34,65] related to the health impacts of ultrafine particles, one more qualitative and one more qualitative, have been organized using this seven step procedure [37], which has proven to work well for these two elicitations.

We have discussed how the types of uncertainties considered, the intended use of the elicited information, and the resources available affect the design and execution of the seven step procedure. First, the type of uncertainty to be elicited can be characterized using an uncertainty typology in the first step. This characterisation subsequently affects choices about the types of experts to invite, and the format of the questions and elicitation session (steps 2, 3 and 4). All of these may greatly differ if the uncertain information to be obtained is qualitative or quantitative, subjective or objective, controversial or undisputed, mono- or multidisciplinary, etc. Second, the intended use of the elicited information influences the design of the expert elicitation. If the information is to be used in the issue framing phase of an assessment, a multidisciplinary panel might be needed (step 3). Expert elicitation may then be used to structure a problem, identify its boundaries, explore different viewpoints or set a strategy for assessment. Such an expert panel usually engages in a rather open discussion, in which consensus is not necessarily strived for. On the other hand, if the estimates are to be used in the execution phase of assessment as a parameter in a mathematical model, then quantification and some aggregation of individual judgements is likely to be required (steps 4, 6 and 7). Finally, the available resources determine the scope of the elicitation (step 2). The seven step procedure presented here is designed for use in IEHIA. It is primarily meant for environmental health related topics and involvement of scientific professionals. Even though the protocol also provides useful information for application outside this primary field, it should not be seen as a universal protocol. As mentioned earlier, for use of expert elicitation in business or court, different procedures and expert qualifications are likely to be necessary [13]. Also, the involvement of people other than scientific professionals requires partly different methods [67,68].

Notwithstanding the potential advantages of using expert elicitation, the method has regularly been subject to criticism and debate. The following issues have been mentioned as sources for criticism against the original Delphi method [8,9], but they are considered to be also applicable to various other expert elicitation methods: low reliability of expert opinions; sensitivity of results to ambiguity in the questionnaire; difficulty in assessing the degree of expertise incorporated into the estimates; incapability of experts to judge the future of events in relation to other developments; and the search for consensus. However, as Cooke [25] points out, many of these issues are not caused by the method as such, but more often by its improper use. These issues are thus also inherent to other (less formal) procedures of review and summarisation of existing scientific evidence. In this paper, we have reviewed how design and documentation of an expert elicitation can overcome, or at least make explicit, most of these limitations.

When there is a high level of value diversity, for example for controversial issues such as genetic modification, some people might be especially sceptical towards the use of expert elicitation. Of course, this is particularly so when the results of the elicitation do not agree with their personal views. In general, people think that their own judgments are less prone to biases than those of people holding opposing views [87,92]. In addition, natural scientists tend to be more sceptical towards using expert elicitation than scientists from other disciplines. In exact disciplines, expert elicitation is not perceived as a reliable or rigorous scientific method, such as those used in empirical studies. Therefore, the results of formal expert elicitation are often considered as being inherently less accurate. Such criticism can often be traced back to a lack of knowledge about formal expert elicitation, or a disproportionate trust in the quality and relevance of empirical data. It can be argued that, most of the time, empirical data also contain many -often implicit- expert judgments [27]. By making such judgments explicit and transparent, as in done in formal expert elicitation, criticism should in fact decrease instead of increase. By designing expert elicitations in a structured way, they somewhat resemble the design of a scientific experiment. This might create some more trust in the results among slightly sceptical (natural) scientists.

Of course there are 'real' limitations to the applicability of expert elicitation. Results provided by expert elicitation represent the synthesis of opinion of a particular group. They can thus not be used to forecast the response of a larger population or even of a different expert panel. Also, experts cannot 'make up' knowledge, when no expertise -in one form or another- is available. Thus, expert elicitation is only limitedly applicable to issues that are extremely uncertain and controversial. In addition, this paper outlines other limitations, such as those related to the effects of heuristics and biases, the criteria about who is considered to be an expert, and the aggregation of results.

## Conclusions

Formal expert elicitation is one of the only options to synthesize scientific knowledge when the development of policy cannot wait for conclusive scientific evidence to become available. The seven step procedure presented in this paper, which draws from several existing protocols for expert elicitation [7,11-21,29,32,33], provides a flexible and practical approach for organizing expert elicitation in the context of integrated environmental health impact assessment. It demonstrates how expert elicitation cannot only provide valuable insights about quantitative uncertainties, but can also be applied to address qualitative issues, such as system boundaries, model structures, and assumptions. We conclude that, despite some known criticism on its validity, formal expert elicitation can support environmental health research in various ways. In view of the great number of uncertain issues in this research area, we believe that formal expert elicitation is a valuable and necessary method to improve our understanding and inform assessments and policies, as well as help prioritise research agendas.

## Abbreviations

AH: Arie Havelaar, personal communication, March 2009; DALY: Disability Adjusted Life Years; DDT: dichlorodiphenyltrichloroethane; EU: European Union; GM: genetic modification/genetically modified; IEHIA: Integrated environmental health impact assessment; Intarese: Integrated Assessment of Health Risks of Environmental Stressors in Europe; IPCC: Intergovernmental Panel on Climate Change; Iqarus: Identification, Quantitative Assessment and Reduction of Uncertainties in burden of disease estimates for environmental Stressors; MKvK: Martin Krayer von Krauss, personal communication, March/April 2009; nm: nanometer; NOx: nitrogen oxides; NUSAP: Numeral, Unit, Spread, Assessment and Pedigree; RIVM: Rijksinstituut voor Volksgezondheid en Milieu (Dutch National Institute for Public Health and the Environment); U.S.: United States.

## Competing interests

The authors declare that they have no competing interests.

## Authors' contributions

PS, ABK and JPS studied the expert elicitation literature and designed the seven step procedure. ABK studied existing environmental health related expert elicitation studies in order to illustrate the procedure, and drafted the main manuscript. EL contributed expertise on the potential use of expert elicitation in IEHIA. All authors read and approved the final manuscript.

## Acknowledgements

We are grateful to prof. dr. ir. Arie Havelaar and dr. Martin Krayer von Krauss for sharing the details of their expert elicitation studies. In addition, we would like to thank prof. dr. Bert Brunekreef for his useful comments to earlier versions of this manuscript, and the three reviewers for their valuable suggestions. The work was funded through the RIVM Strategic Research Project IQARUS and through the EU 6th Framework Project INTARESE.
